# Effectiveness of Green Tea in a Randomized Human Cohort: Relevance to Diabetes and Its Complications

**DOI:** 10.1155/2013/412379

**Published:** 2013-09-12

**Authors:** Naushad Ali Toolsee, Okezie I. Aruoma, Teeluck K. Gunness, Sudhir Kowlessur, Venkatesh Dambala, Fatima Murad, Kreshna Googoolye, Diana Daus, Joseph Indelicato, Philippe Rondeau, Emmanuel Bourdon, Theeshan Bahorun

**Affiliations:** ^1^ANDI Centre of Excellence for Biomedical and Biomaterials Research and Department of Biosciences University of Mauritius, Réduit, Mauritius; ^2^School of Pharmacy and Biomedical Sciences, American University of Health Sciences, Signal Hill, CA 90755, USA; ^3^Cardiac Centre, Sir Seewoosagur Ramgoolam National Hospital, Pamplemousses, Mauritius; ^4^Non-Communicable Diseases Unit, Ministry of Health and Quality of Life, Port Louis, Mauritius; ^5^Department of Biochemistry, Apollo Bramwell Hospital, Moka, Mauritius; ^6^Centre for Clinical Research and Education, Apollo Bramwell Hospital, Moka, Mauritius; ^7^Human Resource Development Council, NG Tower, Ebene, Mauritius; ^8^Department of Occupational Therapy, Touro College of Health Sciences, Bay Shore, NY 11706, USA; ^9^Groupe d'Etude sur l'Inflammation Chronique et l'Obésité, Université de La Réunion, Plate-forme CYROI, Saint Denis, 97400 La Réunion, France

## Abstract

Epidemiological studies have argued that green tea could mitigate diabetes and its complications. This study investigated the phytophenolic profile of Mauritian green tea and its antioxidant propensity. The effect of green tea on the risk factors: waist-hip ratio, glucose level, arterial pressure, antioxidant status, and alanine aminotransferase (ALT) in prediabetics was assessed. The experimental group consumed 3 cups of green tea daily for 14 weeks followed by a 2-week washout period. The control group followed a water regimen. Green tea contained high level of phenolics related to its antioxidant power. Green tea suppressed waist-hip ratio of women from a significant increase and suppressed mean arterial pressure of men and women from a significant decrease after week 14. It reduced ALT level in women by 13.0% (*P* < 0.1) while increasing the antioxidant potential of men and women sera by 2.7% (*P* < 0.1) and 5.1% (*P* < 0.1). The study timescale may have been too short to enable demonstration of effects on fasting plasma glucose and HbA1c outcomes. Green tea regimen could form part of a healthy lifestyle that might ameliorate features of metabolic syndrome and subsequent risks for diabetes and its complications. This trial is registered with ClinicalTrials.gov NCT01248143.

## 1. Introduction

Diabetes has been shown to be a chronic metabolic disorder of multiple aetiologies. The socioeconomic suffering is enormous. Diabetes, characterized by a state of insulin deficiency that leads to a rise in glycemia [1], is commonly classified as insulin-dependent diabetes mellitus (type 1, an autoimmune disease where *β*-cells of the pancreas are affected by the body's defence system) and noninsulin-dependent diabetes mellitus (type 2, a metabolic disorder characterized by insulin resistance and deficiency). Reactive radical species are formed via a number of pathways during hyperglycemia [2–5]. Under normal glycemic condition, glucose primarily undergoes glycolysis and oxidative phosphorylation whereas under hyperglycemic state, glucose could swamp the glycolytic process and hinder glyceraldehyde catabolism, thereby causing fructose-1,6-bisphosphate and glyceraldehyde-3-phosphate to be channelled to other pathways such as enolization and *α*-ketoaldehyde formation; protein kinase C activation; hexosamine metabolism; sorbitol metabolism; dicarbonyl formation and glycation. These combined biochemical pathways play a cardinal role in the pathogenesis of diabetes, whose therapeutic management is argued here to potentially benefit from adjunct inclusion of dietary biofactors in functional beverages [2, 5].

The physiological effects of polyphenol-rich beverages have continued to receive considerable attention as healthful dietary sources of antioxidants [6, 7]. This growing body of evidence advocates the role of green tea or its bioactive polyphenolic compounds in ameliorating features of metabolic syndrome and subsequent risks for type 2 diabetes [8, 9]. Green tea contains important phytochemicals such as catechins, gallic acid, caffeic acid, kaempferol, myricetin, and quercetin [10]. These green tea polyphenols showed antioxidant activities *in vitro *by scavenging reactive radical species and function indirectly as antioxidants through inhibition of redox-sensitive transcription factors; inhibition of “prooxidant” enzymes; and induction of antioxidant enzymes [11, 12]. Several studies have shown that green tea controls glomerular filtration rate, lowers albuminuria, attenuates hypertension, prevents free radical generation in cardiac myocytes, and reduces renal advanced glycation end-product in diabetic nephropathy model rats [13–17]. In this study, the phytophenolic profiles of a green tea infusate were determined, and its antioxidant potential was evaluated using a multiassay approach. The potential modulatory effects of green tea consumption on some selected parameters including waist-hip ratio, glucose level, arterial pressure, antioxidant status, alanine aminotransferase, and lipid profiles were assessed, over a defined period of time, in individuals inclined to develop type 2 diabetes.

## 2. Materials and Methods

### 2.1. Reagents

2,2′-Azobis(2-methylpropionamidine)dihydrochloride (AAPH) was obtained from Sigma-Aldrich, Inc. (St. Louis, MO, USA). Clinical biochemistry reagent kits were purchased from Beckman Coulter Inc. (Brea, CA, USA) and Randox Laboratories Ltd. (Dublin, UK).

### 2.2. Plant Material

*Camellia sinensis* var. *sinensis* (Chinese Jat) was obtained as homogenous green tea bags (finished product) from Bois Chéri Tea Estate (Bois Chéri, Republic of Mauritius). The tea bag, containing approximately 2 g of green tea, was manufactured on November 2010.

### 2.3. Total Phenol Content

Phenol was determined by the method of Singleton and Rossi [18].

### 2.4. Total Flavonoid Content

Flavonoid was determined by the method of Lamaison and Carnet [19].

### 2.5. Total Proanthocyanidin Content

Proanthocyanidin was determined by the method of Porter et al. [20].

### 2.6. High-Performance Liquid Chromatography

#### 2.6.1. Sample Preparation

One green tea bag was infused in 100 mL hot water (100°C) for 6 minutes as described by Wang et al. [21]. The brew was cooled under running tap water and centrifuged at 4000 rpm for 15 minutes at 25°C. For gallic acid, (+)-catechin, (−)-epigallocatechin, (−)-epigallocatechin gallate, (−)-epicatechin gallate, procyanidin B2, and (−)-epicatechin analyses, the supernatant was mixed with absolute methanol in a 2 : 1 supernatant : methanol, v/v ratio prior to HPLC analysis.

Myricetin, kaempferol, and quercetin were identified and quantified in green tea infusate after acid hydrolysis of flavonol conjugates, essentially as follows and using morin as internal standard: the supernatant was mixed with 6 M HCL in a 8 : 1 supernatant : 6 M HCL, v/v ratio and incubated at 85°C for 2.5 hours. After cooling, the hydrolysed green tea supernatant extract was taken up in absolute methanol (3 : 1 v/v, hydrolysed green tea extract : absolute methanol) prior to HPLC analysis.

#### 2.6.2. Chromatographic Conditions

HPLC analysis of the green tea infusate was carried out using a Hewlett Packard 1100 series (Waldbronn, Germany) liquid chromatography system equipped with a vacuum degasser, quaternary pump, autosampler, thermostated column compartment, and diode array detector. After filtration (0.22 *μ*m filter paper) (Millipore Corporation, Bedford, USA), 60 *μ*L of extract was injected into a Zorbax SB-C18 column (4.6 mm internal diameter × 250 mm length, 3.5 *μ*m pore size) (Agilent Technologies, CA, USA). Elution with a flow rate of 0.7 mL/min at 35°C was as follows: 0–30 min, 0–10% B in A; 30–50 min, 10–15% B in A; 50–60 min, 15–25% B in A; 60–90 min, 25–100% B in A; 90–100 min, 100–0% B in A. (Solvent A: acetonitrile/water, 1/9 v/v, pH 2.5; Solvent B: acetonitrile/water, 1/1 v/v, pH 2.5; adjusted with phosphoric acid). The diode array detector was set at 280 nm for the quantitative determination of gallic acid and flavan-3-ol derivatives and at 365 nm for flavonol aglycones. The identification and quantification of these phenolics were determined from retention times and peak areas in comparison with authentic standards. Results were expressed in appropriate standards (mg) per cup (200 mL). Green tea extracts were analysed in triplicate. Calibrated graphs of authentic standards were prepared via appropriate serial dilutions with methanol and filtered (0.22 *μ*m) before use. Calibration graphs were obtained by plotting the peak area of authentic standards versus their known quantities (0.5–4.5 *μ*g).

### 2.7. Sample Preparation for Antioxidant and Free Radical-Induced Hemolysis Assays

Two grams of green tea (equivalent to 1 tea bag) were infused in 200 mL hot water (100°C) for 6 minutes. The green tea brew was cooled under running tap water and filtered through a 0.2 *μ*m nylon filter. The brew was diluted to generate different concentrations of green tea extracts (0.1 mg/mL to 10 mg/mL) for use in antioxidant and free radical-induced hemolysis assays.

#### 2.7.1. Antioxidant Assays

*(1) Superoxide Radical Scavenging Assay.* The superoxide anion radical scavenging activity of green tea was measured according to Nishikimi et al. [22] with slight modifications.

*(2) Nitric Oxide Radical Scavenging Assay.* The nitric oxide radical scavenging activity of green tea was measured according to Garratt [23] with slight modifications.

*(3) 2,2′-Azino-bis (3-Ethylbenzothiazoline-6-sulfonic Acid) Radical Scavenging Assay.* The ABTS^•+^ scavenging activity of green tea was measured according to Henriquez et al. [24] with slight modifications.

*(4) Ferric Reducing Antioxidant Power Assay.* The ferric reducing activity of green tea was measured according to Benzie and Strain [25] with slight modifications.

*(5) 2,2-Diphenyl-1-picrylhydrazyl Radical Scavenging Assay.* The DPPH scavenging activity of green tea was measured according to Sharma and Bhat [26] with slight modifications.

*(6) Ferrous Ion Chelating Assay.* The ferrous ion chelating activity of green tea was assessed according to Stookey [27] with slight modifications.

*(7) Hypochlorous Acid Scavenging Assay.* The hypochlorous acid scavenging activity of green tea was assessed according to Wang et al. [28] and Aruoma and Halliwell [29].

#### 2.7.2. Free Radical-Induced Hemolysis Assay

The antioxidant activities of green tea extracts and human sera, derived from the clinical trial, were evaluated using an assay based on free radical-induced hemolysis [30].

### 2.8. Clinical Trial

#### 2.8.1. Subjects

Three hundred prediabetic Mauritians, who participated in a 2009 nationwide survey that was organized by the Non-Communicable Diseases Unit of the Ministry of Health and Quality of Life, Republic of Mauritius, were selected for the randomized controlled clinical trial (ClinicalTrials.gov Identifier—NCT01248143). The inclusion criteria were individuals at risk of diabetes (fasting plasma glucose ranged from 110 to 126 mg/dL, measured using the hexokinase method); age ranged from 35 to 65 years. The exclusion criteria were smokers or those who have stopped smoking 6 months before the clinical trial; daily alcoholic intake exceeding two standard drinks; postmenopausal women receiving hormone replacement therapy; hypertensive patients (>140/90 mmHg). The clinical trial was approved by the National Ethics Committee of the Ministry of Health and Quality of Life, Republic of Mauritius and endorsed by the Institutional Review Board at Touro College of Health Sciences (Bay Shore, NY, USA). The study was conducted in accordance with the guidelines of the Declaration of Helsinki principles. All subjects gave written informed consent before clinical trial enrolment.

#### 2.8.2. Trial Profile and Study Design

**Figure 1:**
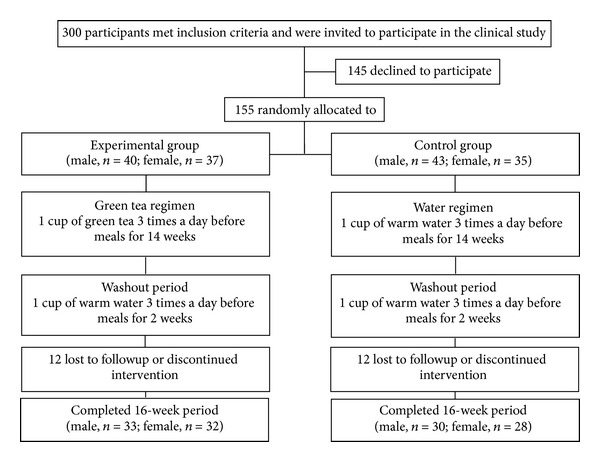
Flow diagram showing trial profile and study design.

The clinical trial was conducted from November 2010 to March 2011 at the Cardiac Centre of the Sir Seewoosagur Ramgoolam National Hospital, Pamplemousses, Republic of Mauritius, over a 16-week period. The experimental group (*n* = 65) consumed one cup of green tea infusate (1 green tea bag infused for 6 min in 200 mL hot water without milk or sugar) three times a day before meals (breakfast, lunch, and dinner) for 14 weeks, followed by a 2-week washout period. The control group (*n* = 58) consumed an equivalent volume of warm water during the 16-week period (Figure 1). The random allocation sequence was generated by a qualified statistician using a random generator that takes into account an unbiased age and gender distribution within each group. A simple randomization approach with blocking for gender was used to allocate subjects into their respective groups. Participants were instructed to maintain their usual daily activities and their former diet during the clinical trial.

#### 2.8.3. Dietary Survey Questionnaires

Participants were contacted twice a week via phone to remind them to keep a record of all food and beverages consumed during the clinical trial. A questionnaire indicating food and beverage items consumed daily during the three main meals was issued to each participant and was collected duly filled after blood sampling exercise. Dietary information provided by the participants enabled the assessment of any possible changes in the diet during the clinical trial. Descriptive statistics of these questionnaires were based on daily mean calorie index and daily lipid/fat ratio [31] observed during the 14-week intervention period and the 2-week washout period.

#### 2.8.4. Anthropometry and Blood Pressure

Anthropometric and blood pressure data were recorded for each participant. Height was measured to the nearest 0.5 cm without shoes using a calibrated stadiometer. Weight was measured without shoes and excess clothing to the nearest 0.1 kg using a calibrated mechanical beam balance. BMI was calculated as weight in kilograms divided by height in square meters. Waist and hip circumference was measured to the nearest 0.5 cm using a dressmaker's measuring tape applied horizontally. Waist girth was measured at the midpoint between the iliac crest and the lower margin of the ribs. Hip girth was recorded as the maximum circumference around the buttocks. Waist-hip ratio was calculated as waist in cm divided by hip in cm. Blood pressure was measured on the right upper arm, in seated and supine position, after resting for five minutes, with an automatic device (Automatic blood pressure monitor Model SEM-1, Omron Healthcare Company, Singapore). Two measurements were taken, with 1 minute interval between them, and the mean of the 2 measurements was calculated. These blood pressure values were used to calculate mean arterial pressure using the standard formula 2/3 diastolic blood pressure + 1/3 systolic blood pressure.

#### 2.8.5. Sample Collection

20 mL of blood was collected, under the guidance of physicians, from each participant fasting for at least 12 hours. The blood was dispensed appropriately into EDTA-K3 tube, sodium fluoride oxalate tube, and plain tubes. Biological samples were kept at 4°C and transported, for analysis, to Apollo Bramwell Hospital, Moka, Republic of Mauritius. Before conducting any assays, all tubes were centrifuged at 3000 rpm for 7 min at 25°C with the exception of EDTA-K3 tube. Human sera from plain tubes were used for free radical-induced hemolysis and clinical biochemistry assay. Whole blood from EDTA-K3 tubes and human plasma from sodium fluoride oxalate tubes were used only for clinical biochemistry assay.

#### 2.8.6. Clinical Biochemistry Assay

Human sera obtained from plain tubes were tested for total cholesterol, LDL, HDL, triglycerides, urea, albumin, creatinine, ferritin, total antioxidant status, aminotransferase aspartate (AST), and ALT. Human plasma obtained from sodium fluoride oxalate tubes were tested for glucose. Whole blood from EDTA-K3 tube was used to determine glycated haemoglobin. Using standardized methods, the automated Beckman Coulter AU480 analyzer was used to quantify all biomarkers, except total antioxidant status which was evaluated by RX Daytona analyzer. All clinical biochemistry assays were completed within 24 h after sample collection. Serum creatinine (nonisotope dilution mass spectrometry traceable), urea, and albumin were used to estimate glomerular filtration rate (eGFR), using the “modification of diet in renal disease” (MDRD) formula [32].

### 2.9. Statistical Analysis

Parametric and nonparametric variables are expressed, after omitting outliers, as mean ± standard deviation and median [interquartile range], respectively. Simple regression analysis was performed to calculate the dose-dependent relationship of green tea extracts in different antioxidant assays. Data derived from antioxidant assays were fitted into appropriate regression model [33] (linear, polynomial order 2 or polynomial order 3) that allowed us to determine AA_50_. Statistical inference was carried out, after omitting outliers, using MedCalc for Windows (version 11.6.0.0; Mariakerke, Belgium). Significant differences over time, within each group, were determined using paired Student's *t*-test, and where data was not normal the nonparametric alternative Wilcoxon test was used. Significant differences over time, between each group, were determined using independent samples *t*-test and where data was not normal the nonparametric alternative Mann-Whitney test was used. The level for establishing significant differences was set at 10% successively. Statistical analyses were also performed to calculate any significant correlations between individual variables, daily mean calorie index, and daily, lipid/fat ratio. Pearson or Spearman rank correlation coefficient was used for normally or nonnormally distributed data sets, respectively. Correlation analyses between individual variables, daily mean calorie index, and daily lipid/fat ratio were carried out separately on week 14 and washout data sets whereby gender stratified data were taken into account. All statistical tests were two-tailed.

## 3. Results

### 3.1. Phytophenolic Composition of Mauritian Green Tea

The Mauritian green tea phytophenolic profile is shown in Table 1. The most prominent phytophenolics, in an unhydrolyzed Mauritian green tea infusate, were ranked according to their quantity per cup (200 mL), in the following decreasing order: procyanidin B2 > (−)-epigallocatechin gallate > (−)-epigallocatechin > (−)-epicatechin gallate > (−)-epicatechin > (+)-catechin > gallic acid. Procyanidin B2 was found to be the most important compound with 496.92 mg per cup (200 mL) while gallic acid was least prominent (1.66 mg per cup).

### 3.2. Antioxidant Capacities of Mauritian Green Tea

The green tea antioxidant profile was characterized using 7 independent assays (Table 2) at concentrations varying from 0.1 to 1 mg/mL. A concentration ranking order indicating 50% antioxidant activity (AA_50_) for the green tea extract in each antioxidant assay was established as follows: ABTS^•+^ scavenging activity (0.01 mg/mL) > ferric reducing activity (0.04 mg/mL) > nitric oxide scavenging activity (0.30 mg/mL) > Fe^2+^ chelating activity (0.33 mg/mL) > HOCL scavenging activity (0.39 mg/mL) > superoxide scavenging activity (0.42 mg/mL) > DPPH scavenging activity (0.52 mg/mL). The data showed that the Mauritian green tea extract had a higher predisposition (*P* < 0.05) to the ABTS^•+^ radical than the DPPH radical. It also revealed a nonsignificant (*P* > 0.05) sensitivity to hypochlorous acid and superoxide anion radical. Moreover, Mauritian green tea had a higher affinity (*P* < 0.05) to reduce Fe^3+^ into Fe^2+^ than to chelate Fe^2+^.

### 3.3. Clinical Trial

**Figure 2:**
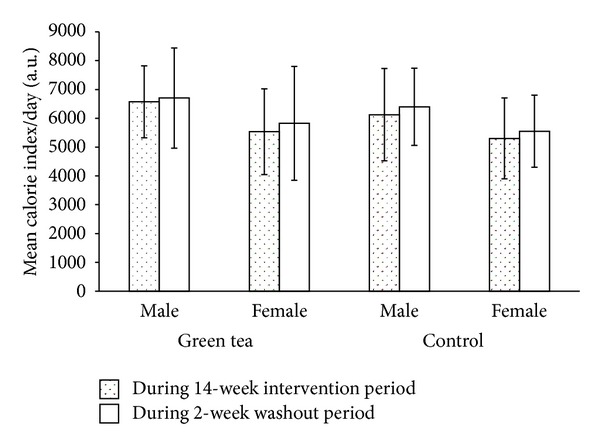
Daily mean calorie index variation for participants under green tea and water regimen during the 14-week intervention period and 2-week washout period in a male and female Mauritian population. Main and error bars represent mean values and standard deviations, respectively, for experimental group (male, *n* = 33; female, *n* = 32) and control group (male, *n* = 30; female, *n* = 28).

**Figure 3:**
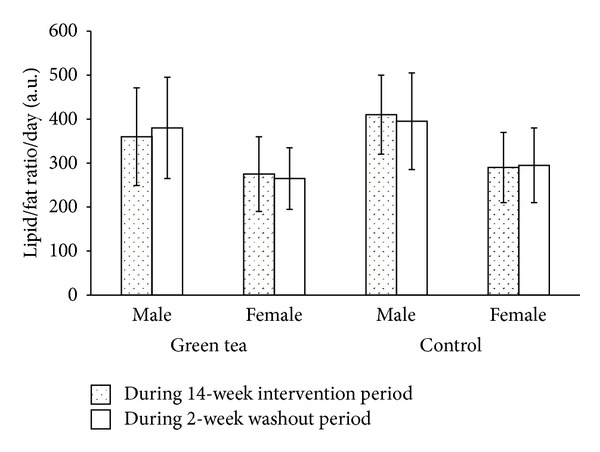
Daily lipid/fat ratio for participants under green tea and water regimen during the 14-week intervention period and 2-week washout period in a male and female Mauritian population. Main and error bars represent mean values and standard deviations, respectively, for experimental group (male, *n* = 33; female, *n* = 32) and control group (male, *n* = 30; female, *n* = 28).

A randomized controlled clinical trial was conducted on humans predisposed to type 2 diabetes. Among the 300 participants that met the inclusion criteria, 155 agreed to enrol in the clinical trial and they were randomly allocated into the experimental and control group. The number of participants at the end of the clinical trial was 123, representing a 20.6% dropout from initial population size. The final experimental and control groups consisted of 65 and 58 participants, respectively. No subject withdrew from the clinical trial due to discomfort or any adverse effects associated with the regimen. The complied dietary survey questionnaires showed that the daily mean calorie index and daily lipid/fat ratio remained relatively constant for both the experimental and control groups in the male and female population during the 14-week intervention period and the 2-week washout period (Figures 2 and 3). No correlation analyses between individual variables, daily mean calorie index, and daily lipid/fat ratio for any group or gender turned out to be significant, nor were there any consistency in the direction of correlations.

The data in Tables 3 and 4 define the anthropometric and biochemical characteristics measured at the beginning of the study, at the end of the 14-week intervention period, and after the 2-week washout period. During the clinical trial, waist-hip ratio, an indicator of obesity, increased significantly (*P* < 0.1) in the female control group whereas the female experimental group did not experience any change on week 14. Mean arterial pressure, defined as the average arterial pressure during a single cardiac cycle, decreased insignificantly on week 14 as indicated by the experimental group whereas male and female control groups endured a critical decline of 3.0% (*P* < 0.1) and 2.6% (*P* < 0.1), respectively.

Based on the findings set out in Table 3, male subjects under green tea regimen showed an insignificant reduction of 3.0% in ferritin concentration after week 14 whereas in male control group its concentration rose considerably by 39.2% (*P* < 0.1). Moreover, the green tea regimen did not affect the fasting plasma glucose of subjects.

eGFR, used to diagnose and monitor kidney function, was estimated using the 6-variable MDRD study equation. During the 16-week period, eGFR increased insignificantly in the male control group. The male experimental group experienced a significant decrease of 7.1% (*P* < 0.1) after week 14, followed by a critical increase of 3.8% (*P* < 0.1) (relative to week 14 value) after the washout period.

The ALT, a transaminase enzyme used to determine liver health, decreased significantly by 13.0% (*P* < 0.1) as illustrated by the female experimental group on week 14, whereas female control group did not show any significant change throughout the clinical trial. The time taken for human sera to delay hemolysis by 50%, representing the antioxidant potential of human sera at the cellular level, increased significantly by 2.7% (*P* < 0.1) and 5.1% (*P* < 0.1) in male and female experimental groups, respectively, after week 14, whereas their corresponding control groups experienced a decrease.

## 4. Discussion

**Figure 4:**
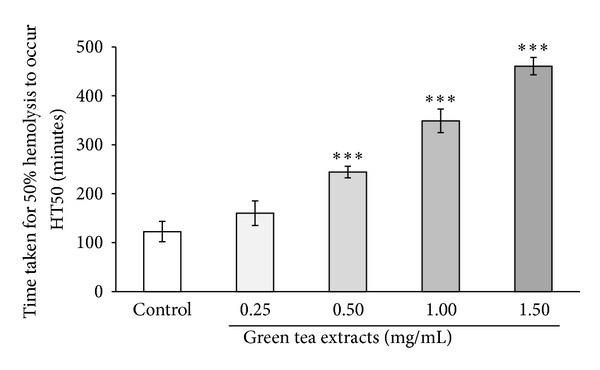
The prophylactic effect of green tea extracts against free radical which might be a causative agent of metabolic syndrome. Main and error bars represent mean value and standard deviation of three independent experiments, respectively. Statistical analyses were performed, using independent samples *t*-test, for multiple comparisons. ***Time taken for 50% hemolysis to occur was significantly different from that of control (*P* < 0.01).

The current management of type 2 diabetes involves a combination of dietary plans, exercise programs, and the use of drugs, such as sulfonylureas and biguanide [34]. Besides these conventional therapies, a growing body of evidence has indicated the role of green tea polyphenols in improving features of metabolic syndrome and subsequent risks for diabetes and its complications [1, 35]. The putative beneficial effects ascribed to green tea polyphenols are partly mediated by their free radical scavenging, antioxidant action, and ability to form complexes with metal ions. The green tea analysed in this study had high levels of total phenols, flavonoids, and proanthocyanidins with a wide range individual compounds that synergistically contributed to the antioxidative propensity of the extract as evidenced by the multiantioxidant analysis data including the free radical-induced hemolysis assay data (Figure 4). Similar observations were made in an earlier report where green and black teas were characterised for their antioxidant functions [36]. The green tea supplementation in this study led to a significant increase in serum antioxidant capacity as measured by the free radical-induced hemolysis assay. This suggests that the oxidative stress condition that can be encountered in diabetes [3, 4] could benefit from green tea consumption. Raza and John [37] have reported that tea catechins can prevent protein degradation by altering subcellular reactive oxygen species production, glutathione metabolism, and cytochrome P450 2E1 activity. Grinberg et al. [38] found that tea polyphenols protect red blood cells against primaquine-induced lysis and H_2_O_2_-induced lipid peroxidation. While it can be further derived from the data in this study that green tea consumption can reinforce *in vivo* antioxidant defences, the potential prophylactic effects of green tea against diabetes and its complications remain to be clearly defined. In the study, the effect of cigarette smoking, excessive alcohol intake, and hormone replacement therapy, on levels of biomarkers measured, were minimised by the exclusion criteria applied to the subject population. Dietary survey questionnaires indicated that daily mean calorie index and daily lipid/fat ratio of the population diet did not change significantly during the 16-week period. The subjects also were not on any medication against diabetes, cardiovascular diseases, or hypertension. Thus, it is not plausible at this stage to suggest whether the effects of green tea regimen could be translated to other dietary factors and medication.

Anthropometric data suggest that green tea regimen for a 14-week intervention period suppressed the significant elevation of waist-hip ratio in the female experimental group. It is of interest to note that in a randomized, double-blind, placebo-controlled clinical trial obese women supplemented with green tea for a 12-week intervention period experienced a marked reduction in waist and hip circumference [39]. The mechanism by which tea or its bioactive components might decrease abdominal obesity may occur at different levels. In the digestive tract, green tea catechins appear to form complexes with lipids and lipolytic enzymes, thereby interfering with the luminal processes of emulsification, hydrolysis, micellar solubilisation, and subsequent uptake of lipids [40]. An increase in 24-hour energy expenditure and fat oxidation in humans has also been attributed to green tea consumption [41].

The consumption of green tea 3 times a day suppressed mean arterial pressure from a significant decrease after week 14, indicating that green tea may promote a good blood flow all around the body and as a consequence prevents ischemic reperfusion injury of end organs. Potenza et al. [42] investigated the metabolic effects of epigallocatechin-3-gallate (EGCG) in spontaneously hypertensive rats. EGCG (200 mg/kg for 3 weeks) significantly reduced systolic blood pressure and enhanced both endothelial function and insulin sensitivity. It has also been shown that epicatechin derivatives from green tea leaves can relax rat mesenteric arteries, probably by inhibiting Ca^2+^ influx and increasing nitric oxide release which has a vasodilatory effect [43, 44].

Green tea consumption during the 14-week period of study did not affect fasting plasma glucose (measured using the glucose oxidase method), nor was the level of the H1Ac impacted. The green tea regimen however prevented impaired fasting glucose from a significant increase as compared to the control group. These findings show the potential role of green tea as a glycemic regulator. The subjects involved in this study are prediabetic who have the potential risk for developing diabetes. Li et al. [45] postulated that green tea catechins might ameliorate insulin resistance by acting as peroxisome proliferator-activated receptor ligands with a dual alpha/gamma agonistic effect. Green tea phytochemicals are also reported to prevent intestinal glucose uptake by inhibiting the sodium-dependent glucose transporter of rabbit intestinal epithelial cells [46]. Many other interventional clinical trials have also illustrated the beneficial effect of green tea against diabetes [47–49]. 

Ferritin, a ubiquitous intracellular protein, stores iron and releases it in a controlled fashion. The amount of ferritin usually reflects the amount of iron *in vivo*. Iron, a redox active transition metal ion, is important for the normal functioning of the human body. However, at high level free ferrous ions react with peroxides to produce hydroxyl radicals, via the Fenton reaction. The formed hydroxyl radicals are highly reactive and can damage cellular components such as proteins, DNA, and lipids. At week 14 of the green tea regimen, the ferritin of male experimental group slightly reduced whereas the ferritin of male control group increased considerably by 39.2% (*P* < 0.1). A plausible explanation for our results is that iron-phenol chelates might form in the lumen during digestion resulting in lower iron absorption [50]. Interestingly, Kim et al. [51] also showed that epigallocatechin-3-gallate inhibit nonheme iron absorption by reducing basolateral iron exit, possibly through formation of a nontransportable polyphenol-iron complex. 

Daily consumption of green tea has been often associated with a lifestyle which may support healthiness and longevity. However, the hepatotoxicity of green tea has been put into question and led to the recent publication of a systematic review of the safety of green tea extracts by the United States Pharmacopeia [52]. In our study, the consumption of green tea three times a day before meals did not show any hepatotoxic effect. Consistent with this outcome is the interventional studies involving supplementation with 714 mg green tea polyphenol daily to healthy men for 3 weeks and supplementation of 500 mg green tea polyphenol daily to postmenopausal osteopenic women for 24 weeks not causing any adverse effects on liver and kidney function, as determined by blood test parameters [53, 54].

Chronic kidney disease is recognized public health problem and eGFR has been considered to be a good indicator to evaluate renal function. Most clinicians claimed that it is difficult to estimate glomerular filtration rate, only from serum creatinine concentration, as its accuracy is affected by other factors [55]. To circumvent these limitations, several formulas have been designed to estimate glomerular filtration rate not only from serum creatinine concentration but also from age, body size, gender, serum albumin concentration, and serum urea concentration [56]. The 6-variable MDRD equation has been used. eGFR of male experimental group decreased significantly on week 14, and after discontinuing the green tea regimen for a 2-week washout period, a critical increase of 3.8% (relative to week 14 value) in eGFR was noticed. Current guidelines define chronic kidney disease as a glomerular filtration rate less than 60 mL/min per 1.73 m^2^ for 3 months or more [57, 58]. On the contrary, the findings in this study showed that the green tea regimen decreased eGFR to a concentration which was still greater than the cutoff point associated with kidney damage. The clinical trial also showed that the green tea regimen is not associated with any side effects or toxicity at the recommended level of intake and revealed prophylactic effects such as antiobesity. Relative to water regimen, the green tea regimen prevents waist-hip ratio of subjects from a significant increase during the intervention period. It significantly increases the antioxidant capacity of serum.

In conclusion, the clinical trial is acknowledgeable to certain limitations. It was not possible for all subjects to maintain their normal diet. Their cooking mode and the amount of food consumed by each subject were not known. In the future, a more detailed dietary survey questionnaire should be used to ensure that the prescribed regimen is being properly followed. The protocol for using 3 cups of green tea was initially based on the total phenolic content corresponding to 235 mg gallic acid equivalent/cup, thus bringing the ingestion to 705 mg of total phenols/day. This was consistent with the literature data [53, 54] and our previous studies using black tea [7, 59] which have shown significant changes in cardiovascular disease clinical parameters with 267 mg of total phenols/day. In the light of the data obtained in this study, a greater intervention period and a consumption of more than 3 cups per day could eventually be envisaged for a more significant modulation of the clinical markers. Nonetheless, more in-depth research is warranted to corroborate these potential benefits in a large multinational cohort [60]. The green tea regimen could form part of a healthy lifestyle that might ameliorate features of metabolic syndrome and subsequent risks for individuals with the potential propensity to develop type 2 diabetes. The mechanisms accounting for the beneficial healthy effects of green tea merit further exploration at the molecular level.

## Figures and Tables

**Table 1:** Mauritian green tea phytophenolic data.

Phytophenolic of unhydrolyzed Mauritian green tea/mg per cup	
(200 mL)	
Total phenol	235.13 ± 30.89^a^
Total flavonoid	7.52 ± 0.97^b^
Total proanthocyanidin	7.89 ± 0.63^b^
Procyanidin B2	496.92 ± 20.08^c^
(−)-Epigallocatechin gallate	234.68 ± 12.08^d^
(−)-Epigallocatechin	205.12 ± 25.42^e^
(−)-Epicatechin gallate	28.46 ± 1.80^f^
(−)-Epicatechin	24.42 ± 2.08^g^
(+)-Catechin	13.90 ± 2.20^h^
Gallic acid	1.66 ± 0.16^i^

Phytophenolics in hydrolyzed Mauritian green tea/mg per cup	
(200 mL)	

Quercetin	1.50 ± 0.16^j^
Myricetin	1.48 ± 0.14^j^
Kaempferol	0.90 ± 0.08^k^

Data are expressed as mean ± standard deviation (*n* = 3). Statistical analyses were performed, using independent samples *t*-test, for multiple comparisons. Different alphabetical superscripts between rows represent significant differences between mean phytophenolic contents (*P* < 0.05). Free flavonoids were not detected in the green tea extract; however, after hydrolysis, HPLC analysis showed that quercetin, kaempferol, and myricetin were the main flavonol aglycones present in the following decreasing order: quercetin > myricetin > kaempferol.

**Table 2:** Antioxidant activities of Mauritian green tea using a multiassay approach.

Antioxidant assays	AA_50_ (mg/mL)
ABTS radical scavenging assay	0.01 ± 0.001^a^
FRAP assay	0.04 ± 0.01^b^
Nitric oxide radical scavenging assay	0.30 ± 0.01^c^
Ferrous ions chelating assay	0.33 ± 0.03^c,d^
HOCL scavenging assay	0.39 ± 0.03^d,e^
Superoxide radical scavenging assay	0.42 ± 0.01^e^
DPPH radical scavenging assay	0.52 ± 0.01^f^

Data are expressed as mean ± standard deviation (*n* = 3). Statistical analyses were performed, using independent samples *t*-test, for multiple comparisons. Different alphabetical superscripts between rows represent significant differences between mean AA_50_ (*P* < 0.05). AA_50_ was defined as Mauritian green tea concentration that revealed a 50% antioxidant activity.

**Table 3:** Characteristics of male participants (*n*) at baseline, after 14-week intervention period, and after 2-week washout period.

Variables	Male green tea group				Male control group			
	*n *	Baseline	Week 14	Washout	*n *	Baseline	Week 14	Washout
Age (year)	33	48.9 ± 6.9			30	48.1 ± 7.9		
Weight (kg)	33	70.8 ± 13.6	71.6 ± 13.5	71.4 ± 13.8	30	75.3 ± 13.3	75.9 ± 13.6	75.4 ± 13.4
BMI (kg/m^2^)	33	24.67 ± 3.69	24.97 ± 3.70 (1.2)*	24.87 ± 3.78 (0.8)*	30	26.28 ± 4.76	26.46 ± 4.79 (0.7)*	26.23 ± 4.71 (−0.2)
Waist-hip ratio	33	0.91 ± 0.06	0.90 ± 0.06 (−0.8)	0.89 ± 0.05 (−1.8)*	30	0.92 ± 0.05	0.91 ± 0.06 (−0.8)	0.90 ± 0.04 (−1.7)*
MAP^a^ (mmHg)	33	93.49 ± 9.48	92.85 ± 9.06 (−0.7)	92.67 ± 10.59 (−0.9)	29	95.62 ± 8.70	92.76 ± 8.73 (−3.0)*	92.29 ± 8.88 (−3.5)*
Glucose (mg/dL)	30	89.70 ± 11.18	90.47 ± 11.65 (0.9)	92.87 ± 9.45 (3.5)	27	90.56 ± 10.35	87.56 ± 11.66 (−3.3)	89.15 ± 11.18 (−1.6)
HbA1c (%)	30	6.00 [5.70–6.20]	5.90 [5.70−6.20]	6.05 [5.80–6.30]*	28	6.00 [5.70–6.20]	5.90 [5.60–6.10]*	5.95 [5.70–6.10]
Cholesterol (mg/dL)	32	218.75 ± 40.39	195.91 ± 34.52 (−10.4)*	199.19 ± 32.72 (−8.9)*	29	215.45 ± 40.01	184.86 ± 35.34 (−14.2)*	194.10 ± 42.06 (−9.9)*
Triglycerides (mg/dL)	31	125.42 ± 54.97	127.77 ± 55.66 (1.9)	115.55 ± 48.38 (−7.9)	26	124.96 ± 47.71	115.58 ± 41.15 (−7.5)	108.04 ± 38.81 (−13.5)*
LDL (mg/dL)	32	145.28 ± 33.72	131.47 ± 28.53 (−9.5)*	134.94 ± 26.89 (−7.1)	27	147.89 ± 37.34	128.26 ± 26.97 (−13.3)*	136.44 ± 35.99 (−7.7)
HDL (mg/dL)	31	48.58 ± 10.92	42.81 ± 11.05 (−11.9)*	44.00 ± 10.31 (−9.4)*	28	45.54 ± 9.41	38.89 ± 8.30 (−14.6)*	40.86 ± 9.26 (−10.3)*
LDL/HDL	31	3.11 ± 0.85	3.22 ± 0.75 (3.5)	3.22 ± 0.77 (3.5)	26	3.38 ± 1.03	3.29 ± 0.62 (−2.7)	3.41 ± 0.76 (0.9)
Ferritin (ng/dL)	29	100.45 ± 72.68	97.41 ± 65.48 (−3.0)	98.97 ± 65.49 (−1.5)	26	80.04 ± 47.12	111.42 ± 75.86 (39.2)*	107.65 ± 72.83 (34.5)*
Albumin (g/dL)	29	4.50 ± 0.23	4.43 ± 0.20 (−1.4)	4.48 ± 0.20 (−0.5)	27	4.50 ± 0.23	4.41 ± 0.23 (−2.1)*	4.47 ± 0.20 (−0.8)
Serum creatinine (mg/dL)	31	1.10 [1.00–1.20]	1.10 [1.10–1.20]	1.10 [1.00–1.20]	27	1.10 [1.10–1.20]	1.10 [1.00–1.20]	1.10 [1.00–1.20]
Urinary creatinine (mg/dL)	29	131.52 ± 65.76	188.17 ± 94.65 (43.1)*	170.66 ± 76.81 (29.8)*	29	138.31 ± 70.17	200.38 ± 64.94 (44.9)*	197.38 ± 80.27 (42.7)*
Urea (mg/dL)	31	14.42 ± 4.66	14.65 ± 3.89 (1.6)	15.48 ± 3.94 (7.4)	27	13.63 ± 2.62	13.85 ± 3.59 (1.6)	12.52 ± 2.64 (−8.2)*
eGFR^b^ (mL/min per 1.73 m^2^)	29	82.62 ± 16.73	76.74 ± 9.00 (−7.1)*	79.64 ± 11.26 (−3.6)	27	81.92 ± 14.24	84.24 ± 13.79 (2.8)^#^	85.55 ± 15.35 (4.4)
AST^c^ (IU/L)	29	26.35 ± 6.22	26.41 ± 5.11 (0.3)	26.79 ± 5.54 (1.7)	28	27.11 ± 7.37	26.93 ± 7.35 (−0.7)	27.11 ± 7.21 (0.0)
ALT^d^ (IU/L)	32	29.09 ± 16.64	27.06 ± 13.45 (−7.0)	27.81 ± 11.39 (−4.4)	28	28.25 ± 12.99	27.54 ± 11.33 (−2.5)	28.61 ± 12.25 (1.3)
TAS^e^ (mmol/L)	29	1.60 ± 0.15	1.75 ± 0.11 (9.5)*	1.92 ± 0.14 (20.1)*	28	1.62 ± 0.13	1.72 ± 0.10 (6.2)*	1.92 ± 0.17 (18.6)*
Human sera HT50 (minutes)	28	165.5 ± 25.7	170.0 ± 27.2 (2.7)*	173.4 ± 26.5 (4.8)*	28	181.4 ± 31.3	176.1 ± 25.5 (−2.9)	178.0 ± 29.1 (−1.9)

Parametric variables are expressed as mean ± standard deviation, whereas nonparametric variables are expressed as median [interquartile range]. Percentage changes relative to baseline means are indicated in round brackets. *Mean or median value is significantly different from that of baseline (*P* < 0.1) (compare within row and within group). ^#^Mean or median value is significantly different from that of week 14 (*P* < 0.1) (compare within row and between group). ^a^Mean arterial pressure; ^b^estimated glomerular filtration rate; ^c^aminotransferase aspartate; ^d^aminotransferase alanine; ^e^total antioxidant status.

**Table 4:** Characteristics of female participants (*n*) at baseline, after 14-week intervention period, and after 2-week washout period.

Variables	Female green tea group				Female control group			
	*n *	Baseline	Week 14	Washout	*n *	Baseline	Week 14	Washout
Age (year)	32	49.3 ± 6.6			28	46.9 ± 8.4		
Weight (kg)	32	61.3 ± 12.2	61.9 ± 12.4	61.7 ± 12.4	28	63.2 ± 9.8	63.6 ± 9.8	63.7 ± 9.9
BMI (kg/m^2^)	31	25.02 ± 3.66	25.17 ± 3.59 (0.6)	25.07 ± 3.53 (0.2)	28	26.64 ± 3.29	26.68 ± 3.34 (0.1)	26.74 ± 3.35 (0.4)
Waist-hip ratio	31	0.82 ± 0.05	0.82 ± 0.06 (0.0)	0.84 ± 0.06 (2.0)*	27	0.83 ± 0.05	0.84 ± 0.05 (1.8)*	0.85 ± 0.03 (2.4)*
MAP^a^ (mmHg)	31	88.80 ± 6.63	87.74 ± 9.15 (−1.2)	88.59 ± 9.16 (−0.2)	27	88.94 ± 8.44	86.63 ± 7.58 (−2.6)*	86.85 ± 6.96 (−2.4)
Glucose (mg/dL)	32	94.41 ± 12.10	94.84 ± 13.47 (0.5)	92.69 ± 10.66 (−1.8)	28	91.50 ± 9.70	95.21 ± 10.62 (4.1)*	91.21 ± 11.69 (−0.3)
HbA1c (%)	30	6.10 [5.90–6.50]	5.95 [5.80–6.30]	6.10 [5.90–6.40]	25	6.00 [5.78–6.10]	5.90 [5.80–6.20]	6.00 [5.88–6.30]*
Cholesterol (mg/dL)	31	209.87 ± 31.81	199.77 ± 34.79 (−4.8)	208.13 ± 37.11 (−0.8)	28	205.32 ± 47.52	188.79 ± 42.14 (−8.1)*	194.04 ± 32.34 (−5.5)*
Triglycerides (mg/dL)	31	118.55 ± 50.73	110.90 ± 51.29 (−6.4)	124.90 ± 74.12 (5.4)	25	93.68 ± 33.53	102.08 ± 35.40 (9.0)	103.16 ± 45.48 (10.1)
LDL (mg/dL)	30	140.40 ± 25.68	129.33 ± 25.51 (−7.9)*	138.30 ± 30.03 (−1.5)	27	135.22 ± 38.55	116.63 ± 37.05 (−13.7)*	128.82 ± 26.20 (−4.7)
HDL (mg/dL)	31	50.36 ± 10.24	48.42 ± 8.90 (−3.8)*	49.55 ± 9.20 (−1.6)	28	52.07 ± 15.29	49.36 ± 13.69 (−5.2)*	49.14 ± 12.36 (−5.6)*
LDL/HDL	29	2.92 ± 0.83	2.76 ± 0.66 (−5.5)	2.90 ± 0.81 (−0.7)	27	2.84 ± 1.25	2.51 ± 0.85 (−11.6)	2.75 ± 0.77 (−3.2)
Ferritin (ng/dL)	29	44.03 ± 36.95	49.17 ± 36.75 (11.7)	50.55 ± 45.01 (14.8)	26	42.73 ± 42.05	40.04 ± 35.60 (−6.3)	45.39 ± 41.97 (6.2)
Albumin (g/dL)	29	4.39 ± 0.26	4.35 ± 0.20 (−0.9)	4.45 ± 0.32 (1.3)	25	4.38 ± 0.16	4.28 ± 0.19 (−2.3)*	4.32 ± 0.19 (−1.4)
Serum creatinine (mg/dL)	31	0.90 [0.83–1.00]	0.90 [0.80–1.00]	0.80 [0.80–0.98]*	28	0.90 [0.80–0.95]	0.90 [0.80–0.90]	0.80 [0.80–0.90]*
Urinary creatinine (mg/dL)	30	94.47 ± 53.48	111.63 ± 76.63 (18.2)	83.40 ± 61.43 (−11.7)	23	95.35 ± 39.22	104.61 ± 57.90 (9.7)	82.74 ± 51.70 (−13.2)
Urea (mg/dL)	32	11.47 ± 2.92	11.63 ± 3.72 (1.4)	10.84 ± 2.84 (−5.4)	27	10.63 ± 2.78	10.07 ± 2.16 (−5.2)	9.89 ± 2.19 (−7.0)
eGFR^b^ (mL/min per 1.73 m^2^)	28	77.70 ± 16.00	80.61 ± 12.26 (3.7)	82.59 ± 11.75 (6.3)*	27	82.42 ± 13.53	85.16 ± 13.64 (3.3)	89.93 ± 13.65 (9.1)*
AST^c^ (IU/L)	31	23.23 ± 4.92	23.39 ± 4.72 (0.7)	22.36 ± 4.62 (−3.8)	27	21.78 ± 5.36	21.93 ± 4.98 (0.7)	22.56 ± 5.03 (3.6)
ALT^d^ (IU/L)	29	18.00 ± 6.72	15.66 ± 3.69 (−13.0)*	16.21 ± 5.02 (−10.0)*	26	17.39 ± 6.73	16.19 ± 4.67 (−6.9)	16.27 ± 5.34 (−6.4)
TAS^e^ (mmol/L)	29	1.48 ± 0.11	1.66 ± 0.11 (11.9)*	1.77 ± 0.09 (19.6)*	24	1.48 ± 0.11	1.60 ± 0.08 (8.3)^∗#^	1.79 ± 0.15 (21.1)*
Human sera HT50 (minutes)	31	160.0 ± 32.3	168.2 ± 32.3 (5.1)*	165.7 ± 32.4 (3.6)*	26	160.1 ± 33.5	158.6 ± 26.1 (−0.9)	159.7 ± 30.7 (−0.2)

Parametric variables are expressed as mean ± standard deviation, whereas nonparametric variables are expressed as median [interquartile range]. Percentage change relative to baseline means are indicated in round brackets. *Mean or median value is significantly different from that of baseline (*P* < 0.1) (compare within row and within group). ^#^Mean or median value is significantly different from that of week 14 (*P* < 0.1) (compare within row and between group). ^a^Mean arterial pressure; ^b^estimated glomerular filtration rate; ^c^aminotransferase aspartate; ^d^aminotransferase alanine; ^e^total antioxidant status.
